# Bufalin-loaded bovine serum albumin nanoparticles demonstrated improved anti-tumor activity against hepatocellular carcinoma: preparation, characterization, pharmacokinetics and tissue distribution

**DOI:** 10.18632/oncotarget.18800

**Published:** 2017-06-28

**Authors:** Huiqing Zhang, Nian Huang, Geliang Yang, Qing Lin, Yonghua Su

**Affiliations:** ^1^ Changhai Hospital of Traditional Chinese Medicine, Second Military Medical University, Shanghai 200433, China

**Keywords:** Bufalin, bovine serum albumin, nanoparticles, hepatocellular carcinoma

## Abstract

**Objective:**

To prepare and evaluate the liver-targeted drug delivery system of Bufalin with higher liver uptake and stronger antitumor activity against hepatocellular carcinoma.

**Methods:**

Bufalin-loaded bovine serum albumin nanoparticle was prepared by desolvation method, to investigate the *in vitro* release performance and to evaluate the pharmacokinetic and tissue distribution. The antitumor activity against hepatocellular carcinoma was evaluated *in vitro* and *in vivo*, respectively.

**Results:**

The Bufalin-loaded bovine serum albumin nanoparticle with an average particle size of 125.1 nm exhibited a sustained release behavior *in vitro*. The half-life, blood plasma area under the curve and apparent volume of distribution of Bufalin-loaded bovine serum albumin nanoparticle were significantly higher than that of Bufalin, whereas the clearance rate was lower than Bufalin group. The uptake of liver for Bufalin-loaded bovine serum albumin nanoparticle was 352.045 ± 35.665 ng/g while for Bufalin was 164.465 ± 48.080 ng/g (*P <* 0.01) at 5 min. The uptake of tumor for Bufalin-loaded bovine serum albumin nanoparticle was significantly higher than that of Bufalin both at 5 min (50.169 ± 11.708 ng/g, 93.415±13.828 ng/g, *P <* 0.01) and 15 min (43.683 ± 11.499 ng/g, 64.219 ± 17.684 ng/g, *P >* 0.05). Bufalin-loaded bovine serum albumin nanoparticle and Bufalin have similar antitumor activity *in vitro*. The tumor inhibition effect of Bufalin-loaded bovine serum albumin nanoparticle was stronger than that of Bufalin alone *in vivo*.

**Conclusion:**

Bufalin-loaded bovine serum albumin nanoparticle is a promising liver-targeted drug delivery system with higher liver uptake and stronger antitumor activity against hepatocellular carcinoma.

## INTRODUCTION

Hepatocellular carcinoma (HCC), one of the most common malignancies worldwide, is a malignant tumor with the fifth highest incidence and the third highest mortality tumor in the world [1]. Despite the available treatment options for HCC, long-term survival rate of patients with HCC is still far from satisfactory due to a high incidence of recurrence after surgery [2, 3]. Approximately 70% of patients who undergo potentially curative procedures will have recurrent or advanced-stage disease within five years [4]. Thus, effective therapies are required for patients with advanced HCC.

**Figure 1:**
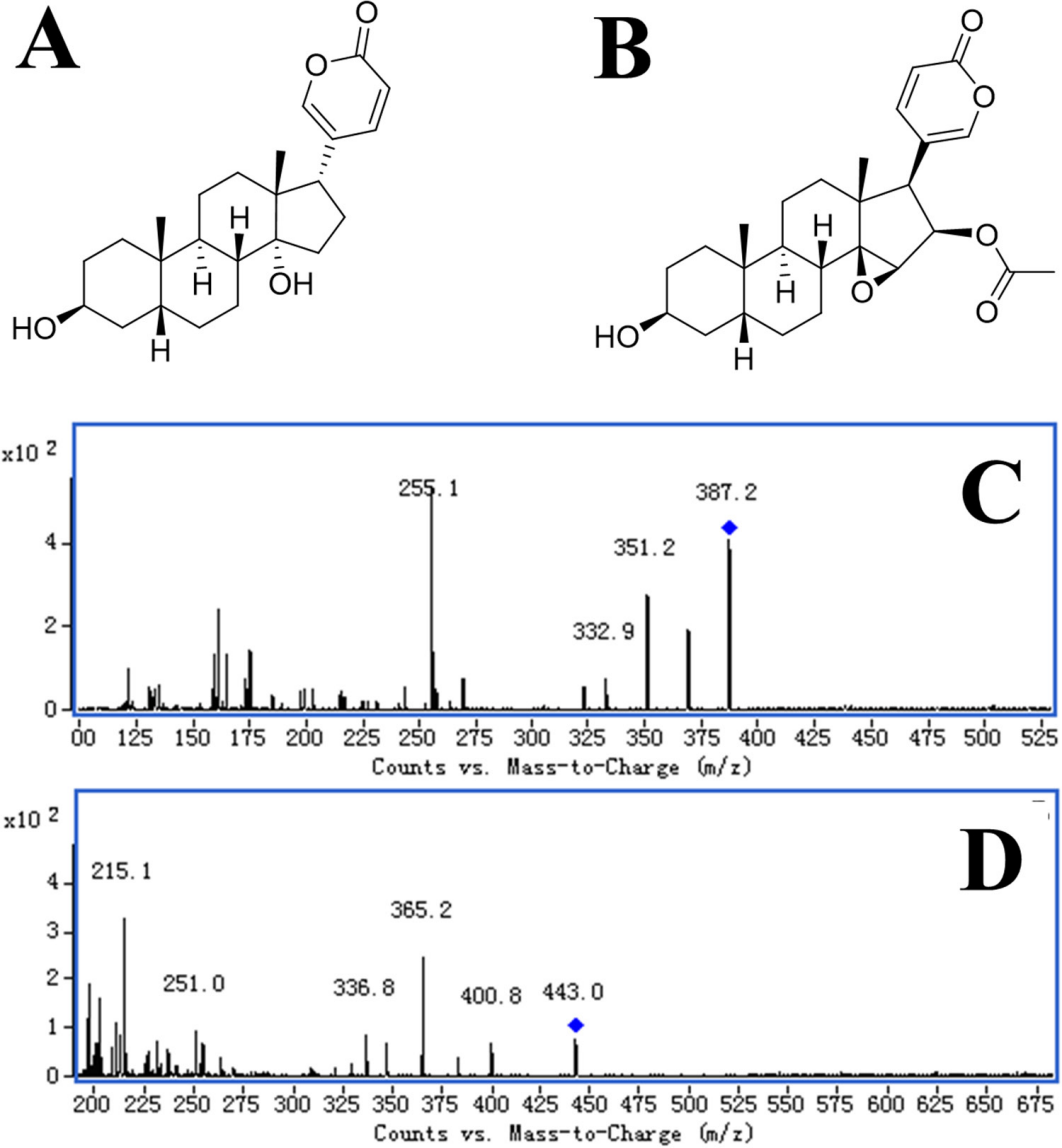
The chemical structure and product ion of Bufalin and Cinobufagin **(A)** Chemical structure of Bufalin; **(B)** chemical structure of Cinobufagin; **(C)** product ion of Bufalin; **(D)** product ion of Cinobufagin.

In recent years, the potential antitumor activity of natural products used in traditional Chinese medicine (TCM) has received much attention [5–7]. Chansu (*Venenum bufonis*), an important antitumor drug obtained from the skin secretions of Bufo bufo gargarizans Cantor or B. melsanostictus Schneider, has been used for thousands of years in China [8, 9]. Huachansu (Cinobufacini), a sterilized hot water extract of dried toad skin, has been approved by the Chinese Food and Drug Administration (ISO9002) and widely used to treat variety of cancers in many China's cancer centers at present, including HCC [10, 11]. Bufalin (3-b,14-Dihydroxy-5-beta-bufa-20,22-dienolide; Figure 1A), a typical digoxin-like immunoreactive component, is the primary bioactive ingredient of Chansu, dried toad skin and Huachansu. A large number of studies have shown that Bufalin could produce a marked anti-tumor effect in different tumors [12–17]. Multiple anti-tumor activities of Bufalin have been recognized, such as inhibition of cell proliferation, induction of cell differentiation, induction of apoptosis, disruption of cell cycle, inhibition of cancer angiogenesis and reversal of multi-drug resistance in cancer cells [8, 12, 14–16, 18, 19]. However, insolubility in water, toxicity, fast metabolize rate and short elimination half-life limit its wide clinical application [20]. It is urgent to develop a drug delivery system that could overcome the above problem of Bufalin while maintaining or enhancing potency.

Nanoparticulate drug delivery system is making a significant contribution to the improvement of drug delivery in cancer [21, 22]. Targeted nanoparticulate drug delivery systems, especially biodegradable nanoparticles, provides opportunities to meet these existing challenges, with the advantages of improved pharmacokinetics, favored tumor accumulation, and reduced side effects [23–25].

Albumin has been used as a versatile protein carrier to fabricate nanoparticles for drug delivery due to its nontoxic, non-immunogenic, biocompatible and biodegradable properties [26]. Bovine serum albumin (BSA) is extensively used for drug delivery because of its abundance, low cost, ease of purification, unusual ligand binding properties and its wide acceptance in the pharmaceutical industry [27].

Bufalin-loaded bovine serum albumin nanoparticles have been firstly prepared by our research group with national invention patent (China, No. 00510110061.4). In this study, we have prepared Bufalin-loaded bovine serum albumin nanoparticles (Bufalin-BSA-NP) by desolvation method and evaluated its effect on antitumor, pharmacokinetic and bio-distribution.

## RESULTS

### Preparation and characterization of Bufalin-BSA-NP

**Figure 2:**
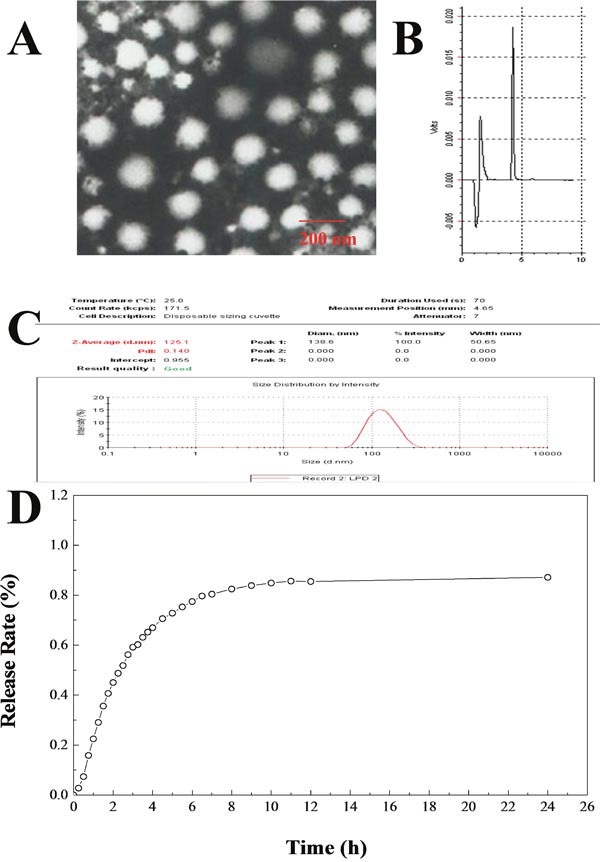
Characterization and physicochemical properties of Bufalin-BSA-NP **(A)** The transmission electron microscope photograph of Bufalin-BSA-NP; **(B)** HPLC results of Bufalin-BSA-NP; **(C)** size distribution by intensity of Bufalin-BSA-NP; **(D)** release curve of Bufalin-BSA-NP *in vitro*.

In this study, the Bufalin-BSA-NP was prepared by desolvation method and its particle morphology was screened by transmission electron microscope (TEM). As shown in Figure 2A, Bufalin-BSA-NP had a uniform spherical morphology with the diameter of 50-250 nm. Quantative analysis of drug loading and encapsulation efficiency of Bufalin-BSA-NP and determination were finished by RP-HPLC method. The retention time of Bufalin is about 4 min, which can be easily identified (Figure 2B). Based on the results, the linear equation of concentration of Bufalin sample (X) and peak area (Y) can be expressed as: Y = −431.79 + 17658.04 X, r > 0.9999. Satisfactory linear relationship can be observed in the range of 2.5-50 *μ*g/ml. The encapsulation efficiency is 76.02% and drug-loading rate is 12.62%. The mean particle size and polydispersity index of prepared Bufalin-BSA-NP were determined to be 125.1 nm and 0.140, respectively (Figure 2C). Zeta potential of the reconstituted Bufalin-BSA-NP was determined to be −19.24 mV. *In vitro* release of Bufalin-BSA-NP was metered by balance dialysis with semi-permeable membrane dialysis bag. The release curve of Bufalin-BSA-NP is shown in Figure 2D. The first 3 h belongs to burst release stage with an accumulative release of 59.16% and then a much slower release rate appears. After 6-8 h, the accumulative release rate increased to 100%, which indicates that the Bufalin-BSA-NP has excellent controlled release function *in vitro*. In addition, about 7.34% of drug has been released after 0.5 h, which compiles to the Pharmacopoeia of China (< 40%). *In vitro* release equation of Bufalin-BSA-NP can be expressed as: lnln [1/(1-Q)] = −1.4092+ 0.9581 lnt, (r = 0.9535), which is determined by Weibull equation. Bufalin is insoluble in water, while Bufalin-BSA-NP has good solubility. The aqueous solution of Bufalin-BSA-NP is light yellow and clear liquid.

### Acute toxicity of Bufalin-BSA-NP

**Figure 3:**
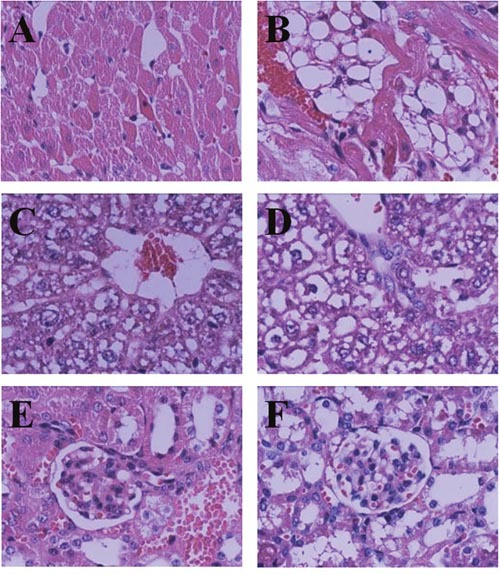
Pathology changes in HE staining (HE×400) **(A)** 8mg/kg Bufalin-BSA-NP Heart; **(B)** 8mg/kg Bufalin Heart; **(C)** 8mg/kg Bufalin-BSA-NP Liver; **(D)** 8mg/kg Bufalin Liver; **(E)** 8 mg/kg Bufalin-BSA-NP kindey; **(F)** 8 mg/kg Bufalin kindey.

The LD_50_ of Bufalin-BSA-NP was 4.377 mg/kg (relative concentration of Bufalin), and its 95% confidence interval (CI) was 3.765-5.099 mg/kg, while that of Bufalin was 2.739 mg/kg (95% CI: 2.351-3.191 mg/kg). Between the groups of Bufalin-BSA-NP and Bufalin, there are no abnormal changes of major organs in naked eyes after the gross anatomy. Results of HE staining pathology showed no obvious pathological changes in liver, heart, spleen, lung, kidneys and brain in the low dose. When the Bufalin group was in 2.25 mg/kg dose, the mice's liver, heart and kidneys appeared oedema, while the Bufalin-BSA-NP group was in 4.23 mg/kg dose appear. When the drug is at high doses, the two different groups of mice's heart, liver, spleen, lung, kidneys, brain and other major organs are all have different degrees of pathological changes, among of the organs, the heart, liver and kidneys manifest the most serious degree, and their pathological changes are similar. In addition, the Figure 3 showed that the Bufalin-BSA-NP caused less damage to heart, liver and kidneys. From the perspective of death in mice caused by the both injection, the death time of Bufalin-BSA-NP group extended obviously than Bufalin group. The group of the highest dose of Bufalin turns up fastest death time in 2-3 min after the treatment, while Bufalin-BSA-NP group appears fastest death time in 6-7 min at the same dose.

### Pharmacokinetic study of Bufalin-BSA-NP

**Figure 4:**
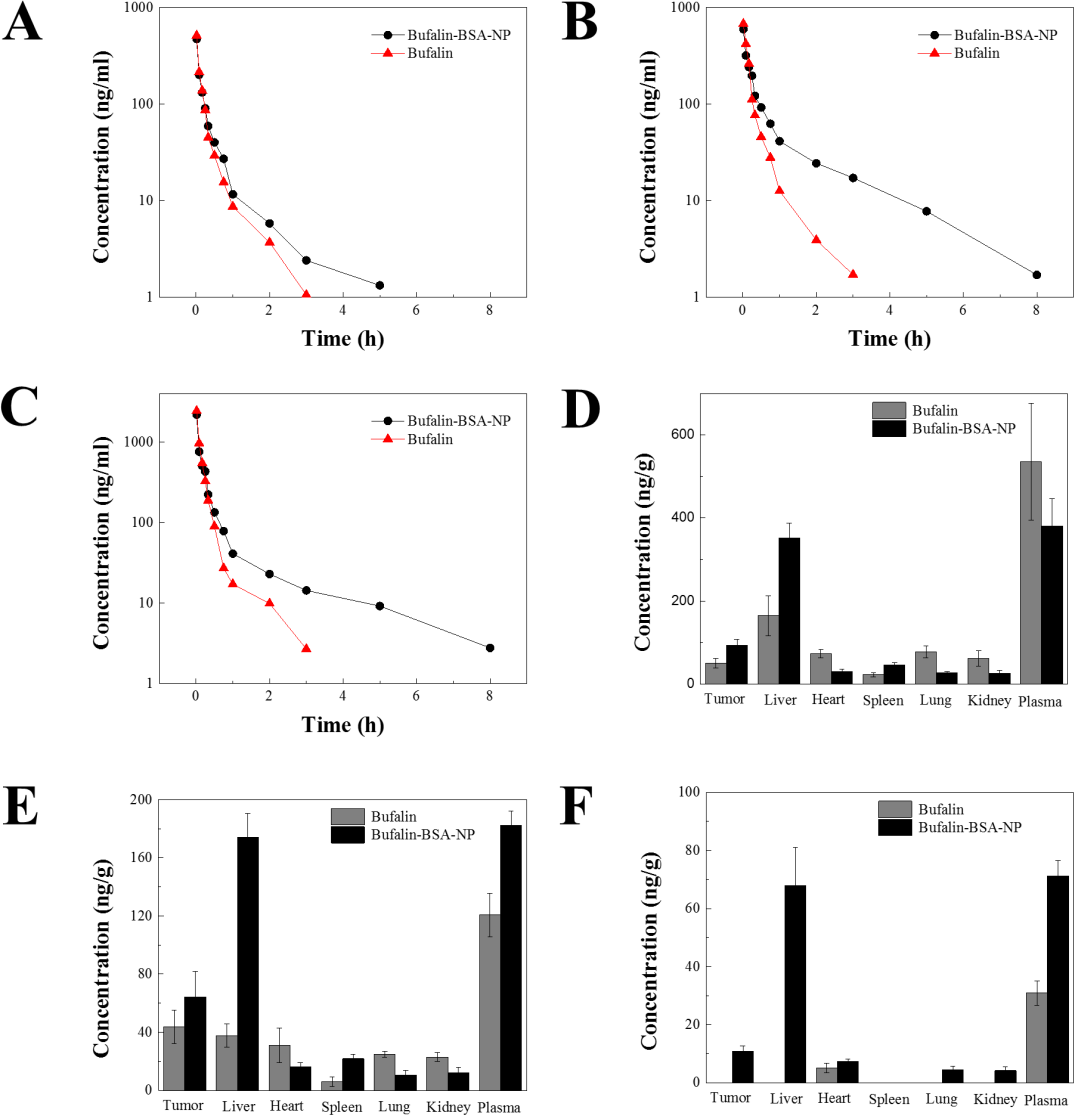
Pharmacokinetic and tissue distribution of Bufalin-BSA-NP *in vivo* **(A, B, C)** Bufalin concentration of Bufalin and Bufalin-BSA-NP at doses of 0.15 (A), 0.3 (B) and 0.6 (C) mg/kg, respectively; **(D, E, F)** tissue distribution of Bufalin and Bufalin-BSA-NP *in vivo* at 5, 15, and 45 min after a single dose of 0.3mg/kg. Values are expressed as mean ± standard error of mean; *n* = 5.

Six groups of Wistar rats were treated with Bufalin-BSA-NP and Bufalin at a single dose of 0.6, 0.3 and 0.15mg/kg, respectively. Figure 4A, 4B, and 4C showed the Bufalin plasma concentration-time curves after intravenous administration of different formulations. Compared with Bufalin-BSA-NP groups, Bufalin was quickly removed from the circulating system. No Bufalin was detected after 4 h, while Bufalin-BSA-NP was still present in the plasma until 10h post-injection of Bufalin-BSA-NP and Bufalin. The nanoparticles showed a markedly delayed blood clearance with higher Bufalin concentration at later time points. Mean pharmacokinetic parameters of Bufalin-BSA-NP and Bufalin were listed in Figure 4A, 4B, and 4C. The pharmacokinetic parameters were calculated using noncompartmental methods. As could be seen from Table 1, The blood plasma area under the curve (AUC), mean residence time (MRT), half-life and clearance rate of Bufalin-BSA-NP were 1.19-1.81, 2.12-3.61, 2.17-2.94 and 0.55-0.82 times of Bufalin solution, respectively.

**Table 1:** Pharmacokinetic parameters of Bufalin and Bufalin-BSA-NP at doses of 0.15, 0.3 and 0.6 mg/kg

Pharmacokinetic parameters	0.6 (mg/kg)		0.3 (mg/kg)		0.15 (mg/kg)	
	Bufalin	Bufalin-BSA-NP	Bufalin	Bufalin-BSA-NP	Bufalin	Bufalin-BSA-NP
*C*_max_ (ng/mL)	2430.5	2186.2	682.3	599.9	515.6	474.3
t_1/2_ (min)	43.2	127.2	39	93	34.8	75.6
MRT (min)	19.2	68.4	24.6	88.8	24	51
AUC_0-8_ (ng h/mL)	299.7	380.8	126.6	229.6	77.6	92.5
AUC_0-∞_ (ng h/mL)	302.6	389.7	128.22	233.48	78.53	94.98
Vd (L/kg)	0.64	1.76	0.95	1.90	0.76	1.34
CL (L/h kg)	2.00	1.54	2.34	1.29	1.93	1.58

Bufalin-BSA-NP: Bufalin-loaded bovine serum albumin nanoparticle; t_1/2_: half-life of plasma; *C*_max_: maximum concentration; MRT: mean residence time; AUC: area under the curve; Vd: aparent volume of distribution; CL: clearance.

### Tissue distribution of Bufalin-BSA-NP

The concentration of Bufalin in plasma and tissue were investigated after intravenous administration of Bufalin and Bufalin-BSA-NP in Wistar rats bearing Walker-256 transplanted liver cancer. The concentration of Bufalin was shown in Figure 4D, 4E and 4F. As shown in Figure 4D, 4E and 4F, concentration of Bufalin in plasma after injection with Bufalin-BSA-NP was higher than that of Bufalin solution at the later time points, which was in agreement with the result of pharmacokinetics in rats. The level of Bufalin concentration of Bufalin-BSA-NP group was lower than that of Bufalin group in all tissue except the liver, tumor and spleen. Especially, the level of Bufalin concentration of Bufalin group was markedly higher than that of Bufalin-BSA-NP group in liver. The uptake of liver for Bufalin-BSA-NP was 352.045 ± 35.665 ng/g and 164.465 ± 48.080 ng/g for Bufalin (*P <* 0.01) at 5 min. Moreover, the liver uptake of Bufalin-BSA-NP was also higher than that of Bufalin (174.138 ± 16.404 ng/g versus 37.689 ± 7.860 ng/g, *P <* 0.01). This might possibly due to the passive targeting ability of BSA nanoparticles for enhanced permeability and retention (EPR) effect [28]. Furthermore, the uptake of tumor for Bufalin-BSA-NP was significantly higher than that of Bufalin both at 5 min (50.169 ± 11.708 versus 93.415 ± 13.828, ng/g, *P <* 0.01) and 15 min 43.683 ± 11.499 versus 64.219 ± 17.684, *P >* 0.05). Interestingly, the uptake of heart, lung and kidney for Bufalin-BSA-NP were all lower than that of Bufalin. For administration of Bufalin-BSA-NP at 5 min, 72.537 ± 9.916, 77.087 ± 13.965, and 61.396 ± 18.126 ng/g of Bufalin were distributed in liver, heart and kidney, respectively. In contrast, for the same time period of Bufalin, 29.695 ± 6.314, 26.919 ± 3.358, and 25.638 ± 7.657 ng/g of Bufalin were distributed in the above organs, respectively. These results obviously demonstrated that Bufalin-BSA-NP was superior to Bufalin since Bufalin-BSA-NP could deliver more Bufalin to both liver and tumor and reduce the toxicity to heart, lung and kidney.

### Bufalin-BSA-NP demonstrated similar anti-tumor effects to Bufalin *in vitro*

**Figure 5:**
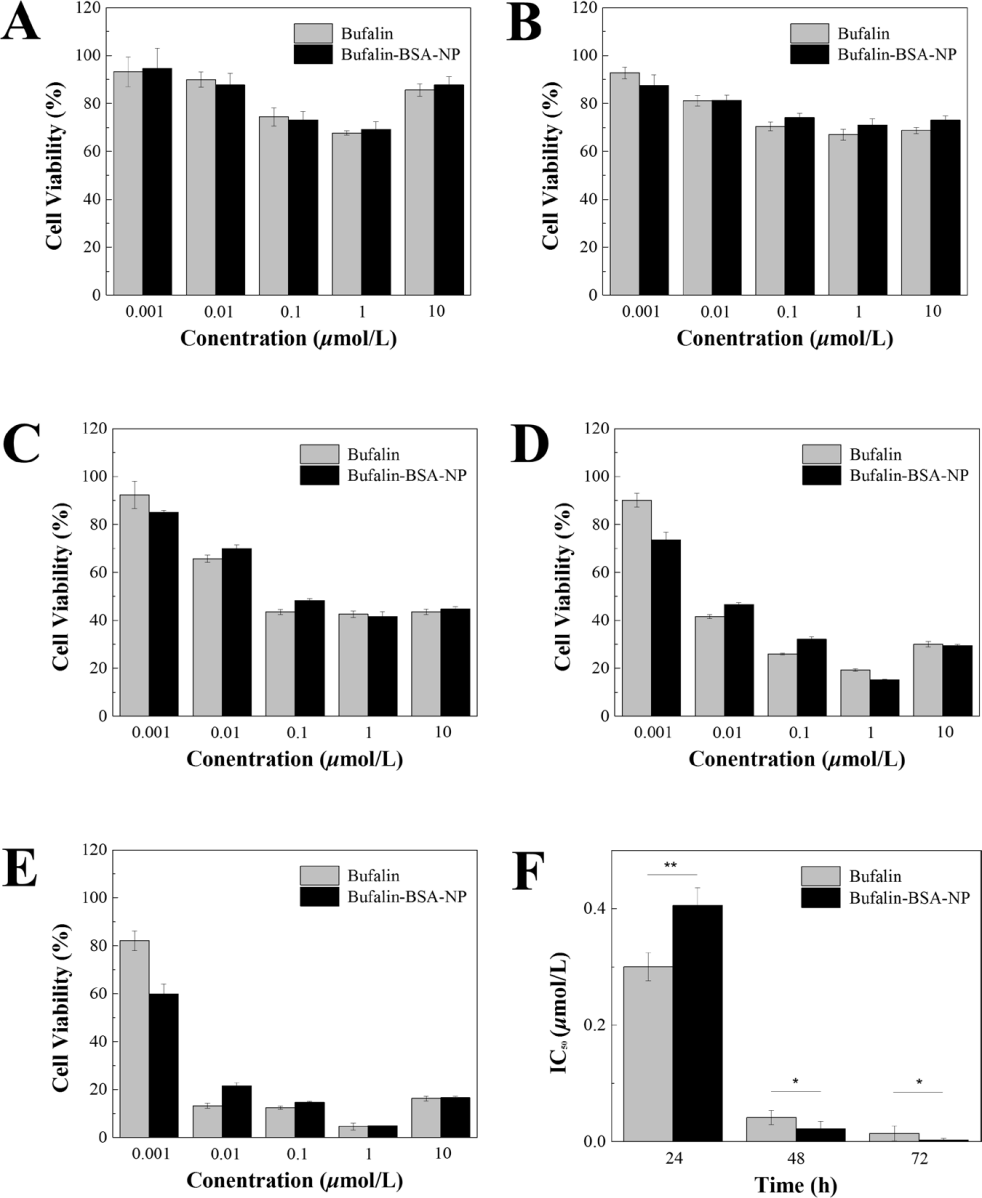
Bufalin-BSA-NP demonstrated similar anti-tumor effects to Bufalin *in vitro* **(A)** The cell viability after sustained action of 6 h; **(B)** the cell viability after sustained action of 12 h; **(C)** the cell viability after sustained action of 24 h; **(D)** the cell viability after sustained action of 48 h; **(E)** the cell viability after sustained action of 72 h; **(F)** half inhibitory concentration (IC_50_) of Bufalin-BSA-NP. Values are expressed as mean ± standard error of mean; *n* = 5. *P < 0.05; ** *P <* 0.01.

As plotted in Figure 5, the cellular inhibition effect of Bufalin-BSA-NP on cell of SMMC-7721 is similar with Bufalin. From analyzing the cell viability, there is a statistical difference on different concentrations or point in time inhibition rate (*P <* 0.01 or *P <* 0.05), but did not reflect obviously the both on the strength of interaction. The inhibition rate to a certain range (10^−6^-10^−8^ mol/L) increases with the increase of concentration. When the concentration of Bufalin-BSA-NP was more than 10^−6^ mol/L levels, each period inhibitory rate did not increase significantly. With Loggt method, geting role for 24 h, IC_50_ of Bufalin-BSA-NP is 1.81×10^−7^ mol/L while that of Bufalin is 1.56×10 mol/L (*P >* 0.05). When for 48 h, IC_50_ of the both are respectively 1.12×10^−8^ mol/L and 2.33 × 10^−8^ mol/L.

### Bufalin-BSA-NP demonstrated more potent anti-tumor effects than Bufalin on hepatocellular carcinoma in nude mice

**Figure 6:**
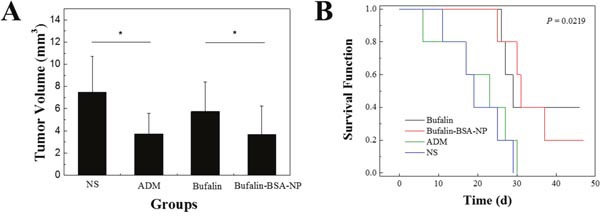
Bufalin-BSA-NP demonstrated more potent anti-tumor effects than Bufalin on hepatocellular carcinoma in nude mice **(A)** Tumor volume of different groups; **(B)** survival analysis of different groups. ADM: adriamycin; NS: normal saline. Values are expressed as mean ± standard error of mean; *n* = 10. *P < 0.05.

In the *in vivo* tumor inhibition test, there were significant differences in the tumor mass in the Bufalin-BSA-NP and Bufalin groups (*P <* 0.05; Figure 6). The difference between each group was also significant (*P<*0.05), suggesting that both Bufalin-BSA-NP and Bufalin alone could suppress cancer of the liver, although the tumor inhibition effect of Bufalin-BSA-NP was stronger than that of Bufalin alone. After treatment, the weight of three groups (Bufalin, Bufalin-BSA-NP and adriamycin) and NS group have significant differences. Among them, the weight of Bufalin and Bufalin-BSA-NP group increased significantly, whereas the weight of normal saline (NS) and adriamycin (ADM) group are significantly reduced. The group of ADM compared with before and after treatment, the weight significantly reduction (*P* < 0.01). Mice skin fold obviously, the group of ADM life status is the worst, and diet, activity is significantly reduced; while Bufalin, Bufalin-BSA-NP, NS group of nude mice living conditions is better, diet, activity is basic normal, and Bufalin and Bufalin-BSA-NP group of weight increase than pre-dose, NS group weight change is not obvious.

Most mice in each group died within 45 d after administration. From Figure 6, compared with NS and ADM groups, the average survival time of the mice with orthotopic transplantation liver cancer model administered Bufalin-BSA-NP and Bufalin have been extended (*P* < 0.05). In addition, the survival time of Bufalin-BSA-NP have lengthened slightly compared with Bufalin group. However, the difference is not statistically significant (*P* > 0.05). Compared with NS group, ADM group does not prolong survival time without statistically significant difference.

## DISCUSSION

In the present study, Bufalin-BSA-NP was successfully prepared as a liver-targeted drug delivery system. *In vitro* release profiles of Bufalin from Bufalin-BSA-NP exhibited a sustained release behaviour. A similar phenomenon was also observed in the *in vivo* pharmacokinetic study.

Compared with Bufalin solution, Bufalin-BSA-NP showed higher AUC value and a prolonged residence of drug in blood circulation. Moreover, higher drug concentration and longer residence time of Bufalin in liver demonstrated that Bufalin-BSA-NP could directly target to liver and reduce the side effects of Bufalin to other organs.

Our results showed that the micellar size obtained in size distribution by intensity was consistent with the results of TEM. Particle size and its distribution are the most widely accepted defining characteristics of nanoparticle-based medicines since particle size can significantly influence the pharmacokinetics, biodistribution, and safety of nanoparticulate drugs [29].

The acute toxicity data indicated that the toxicity of Bufalin-BSA-NP is lower than that of Bufalin. The harmacokinetic behaviour of Bufalin-BSA-NP displayed significantly higher AUC (p50.05) and slower clearance (p50.05) than that of Bufalin solution, indicating that Bufalin-BSA-NP increased the systemic circulation time and a higher amount of Bufalin available for tissue uptake. These results given above were related to the metabolic pattern of Bufalin *in vivo*, which was in agreement with the *in vitro* sustained release of Bufalin from the nanoparticles. When Bufalin solution was injected into the vein, Bufalin was directly exposure to blood, resulting in faster elimination. However, the nanoparticles can serve as reservoirs for Bufalin in blood, which prevent the interactions between Bufalin and the blood components. When Bufalin-BSA-NP was gradually degraded, the Bufalin loaded in it was slowly released into blood, leading to prolonged residence of Bufalin in blood circulation, slower clearance and higher AUC. Therefore, it could be concluded that Bufalin-BSA-NP could improve the availability of Bufalin by prolonging drug retention and more tissue uptake *in vivo*. The pharmacokinetic study showed that the average particle size of Bufalin-BSA-NP was about 125.1 nm. Its half-life, average retention time, AUC and apparent volume of distribution were significantly higher than that of Bufalin group, whereas the clearance rate was lower than Bufalin group. The above results indicated that Bufalin-BSA-NP eliminates in the blood more slowly than Bufalin. And it can maintain blood drug concentration in a long time with sustained release properties *in vivo*.

The results of tissue distribution showed that Bufalin targeting liver tissue is apparent. On one hand, there is high drug concentration in liver tissue. One the other hand, high concentrations of drugs in the liver tissue sustained for a long time. However, the drug content of Bufalin-BSA-NP and Bufalin group in liver tissue were higher than normal liver tumor tissue, the possible reasons are molding and sampling. The cause may be the blood supply of normal liver tissue is greater than that of tumor tissue in liver. This is still a certain difference with the actual clinical pathology of hepatic carcinoma. In addition, the result also showed drug content of Bufalin-BSA-NP in liver tumors is higher than that of Bufalin, which showed Bufalin-BSA-NP have certain targets for liver tumors compared with Bufalin. The experimental results of another phenomenon is worthy of attention is that Bufalin group of drug content is very low in the spleen, but Bufalin-BSA-NP group's drug concentration in the spleen is higher than that of Bufalin. This may be related to the spleen and liver belong to the reticuloendothelial system rich in viscera. Particle size of nanoparticles is easily taken by RES between 100-200 nm.

The results of *in vitro* antitumor activity suggested that the process of preparation of bovine serum albumin nanoparticle did not destroy the antitumor activity of Bufalin. Therefore, Bufalin-BSA-NP and Bufalin have similar antitumor activity *in vitro*.

As illustrated in Figure 6, the average survival time of the mice with orthotopic transplantation liver cancer model administered Bufalin-BSA-NP and Bufalin have been extended (*P* < 0.05). Moreover, there were significant differences in the survival time of Bufalin-BSA-NP group and Bufalin group (34.0 ± 8.4 d versus 31.0 ± 6.5 d, P < 0.01). However, the difference was not statistically significant (*P* > 0.05). Compared with NS group, ADM group did not prolong survival time without statistically significant difference.

The present results thus suggest that Bufalin-BSA-NP may become a promising drug delivery system for hepatocarcinoma targeting therapy.

## MATERIALS AND METHODS

### Materials

Bufalin, Cinobufagin, bovine serum albumin (BSA), 3-(4, 5-dimethyl-2-thiazyl)-2, 5-diphenyl-2H-tetrazolium bromide (MTT), glutaraldehyde and dimethyl sul-foxide (DMSO) were purchased from Sigma-Aldrich (St Louis, MO, USA). Acetonitrile and methyl alcohol were purchased from Fisher Scientific (Pittsburgh, PA, USA). High-glucose Dulbecco's Modified Eagle's Medium (DMEM), Fetal Bovine serum (FBS), Pancreatic enzyme and Ethylene Diamine Tetraacetic Acid (EDTA) were purchased from Hyclone Laboratories (Logan, UT, USA). Adriamycin was purchased from Wanle Pharmacia (Shenzhen, China). Pentobarbital sodium was obtained from Merck Corp (Darmstadt, Germany). All solvents were of high-performance liquid chromatogra-phy grade. All reagents were of analytical grade.

### Preparation of Bufalin-BSA-NP

50 mg bovine serum albumin was dissolved in water while 5 mg Bufalin was dissolved by 95% mlethyl alchohol. Both two types of solution were homogeneous and transparent. Then under pH value of 5-7, Bufalin solution was added into bovine serum albumin aqueous solution. While continuous stirring, specific amount of 95% ethyl alchohol acting as dehydrant was added into the above mixed solution with a constant injection speed of 0.8ml/min. After transparent emulsion formed, cross-linking agent 4% glutaraldehyde was applied to solidify the Bufalin-BSA-NP, then stirring it for 24 hours, Organic solvents such as ethyl alcohol were removed by underpressure evaporation, the remained solution volume was precisely determined.

### Characterization of Bufalin-BSA-NP

Particle morphology was investigated by TEM after negative stained with phosphonic acid wolfram. The average particle size, particle size distribution and Zeta potential of Bufalin-BSA-NP were determined by laser light scattering method. The as prepared Bufalin-BSA-NP emulsion was centrifugated (10,000 rpm/min, 4°C) for 30 min to acquire desalinized Bufalin-BSA-NP in the lower layer and free Bufalin solution in the up layer, of which the amount of Bufalin was measured by reverse-phase high-performance liquid chromatography (RP-HPLC, Hitachi Co., Tokyo) method. Encapsulation efficiency (EE%) and drug loading (DL%) were worked out by following equations: EE% = Bufalin in Bufalin-BSA-NP/total Bufalin × 100%; DL% = Bufalin in Bufalin-BSA-NP/weight of Bufalin-BSA-NP × 100%; where the weight of Bufalin-BSA-NP is equal to albumin plus Bufalin in Bufalin-BSA-NP.

Dialysis bag containing desalinized Bufalin-BSA-NP emulsion was put into 100 ml of phosphate buffer (pH 7.4). The buffer solution was oscillated in thermostatic oscillator (37 ± 1°C, 50 ± 1 shake frequency/min). At a specific time, 1 ml of suspension was taken out and the concentration of Bufalin was then measured with RP-HPLC. Finally, the accumulative release rate could be calculated with the concentration data measured.

### Acute toxicity test

Acute toxicity test of Bufalin-BSA-NP and Bufalin was investigated in Kunming mice (aged 3 weeks, weighing 20 ± 1.0 g), provided by the experimental animal center of the Second Military Medical University (Shanghai, China). This study protocol has been approved by the ethical review boards of Second Military Medical University. Animal welfare and experimental procedures were carried out in accordance with the guide for the care and use of laboratory animals and related ethical regulations of National Research Council's Guide for the use of laboratory animals. The mice were randomly assigned into 14 groups, with five females and five males in each group. Bufalin-BSA-NP and Bufalin were given respectively to the mice at dosages of 8, 5.81, 4.23, 3.09, 2.25, 1.64 and 1.20 mg/kg (relative concentration of Bufalin). All mice were injected in the caudal vein by 0.2 ml and calculated the number of lives lost. The median lethal dose (LD_50_) values were evaluated by Bliss method [30]. The organic tissue of heart, liver, spleen, lung and kidney were collected from dead mice quickly and fixed in 10% (v/v) neutral formalin solution. Twenty-four hours post-injection, all the mice were euthanized and organs were harvested and fixed as stated above. The pathological changes of important organs were studied by hematoxylin-eosin (HE) staining.

### Pharmacokinetic and biodistribution studies

The analyses were performed on an Agilent 1200 series high-performance liquid chromatography (HPLC) and an Agilent 6410 triple-quadrupole mass spectrometer equipped with an electro-spray ionization source (Agilent Technologies, MA, USA). The mobile phase was acetonitrile-water (65:35, v/v) containing 0.1% formic acid. A DIKMA Inertsil ODS-3 column (150 mm×2.1 mm, 5 *μ*m) with an Agilent Zorbax SB-C18 column (100 mm×2.1 mm, 3.5 *μ*m) was used for liquid chromatographic separation. The column was equilibrated and eluted under isocratic conditions with a flow rate of 0.3 mL/min, maintained at 35°C. The sample injection volume was 10 *μ*L and the run time was 2.2min. Quantification was performed in negative multiple reaction monitoring (MRM) of the transitions *m/z* 387.2 → 255.1 for Bufalin (Figure 1A and 1C) and *m/z* 443.2 → 365.1 for the internal standard (IS, Cinobufagin, Figure 1B and 1D). The detection parameters were optimized as follows: drying gas temperature, 350°C; drying gas flow, 10 L/min; nebulizer pressure, 40 psi; capillary voltage, 4000 V; fragmentor voltage, 180 V for Bufalin and 135 V for the IS; collision energy, 22 eV for Bufalin and 18 eV for the IS (Table 2).

**Table 2:** The optimized MRM parameters for Bufalin and Cinobufagin

Group	Precursor ion	Fragmentor energy (V)	Collision energy (eV)	Production
Bufalin	387.2	180	22	255.1
**Cinobufagin**	**443.2**	**135**	**18**	**365.2**

The pharmacokinetic and biodistribution studies were performed on Wistar rats bearing Walker-256 transplanted liver cancer [31]. Walker-256, a rat hepatoma cell line (inoculated with breast cancer cells to the liver tumor), is similar to human liver cancer in blood supply and growth behavior after liver transplantation, and has been widely applied in experimental treatment, metastasis and pharmacokinetic of HCC [32].

Six group of rats for pharmacokinetic were intravenously administered with Bufalin or Bufalin-BSA-NP at different doses (0.6, 0.3 and 0.15 mg/kg), and blood samples were collected from postorbital venous plexus veins at selected time points (1, 5, 8, 10, 15, 20, 30, 45, 60, 120, 180, 300 and 480 min) on same rats. Blood samples were collected in heparinized tubes, and spun down at 3500 rpm/min for 10 min to isolate plasma, and the plasma samples were stored at −80°C until analysis. Two groups of rats for biodistribution were intravenously administered with Bufalin or Bufalin-BSA-NP at dose of 0.3 mg/kg. Organs (liver, heart, lung, spleen, kidney and brain) and tumor tissue were harvested, weighed, rinsed and homogenated in normal saline. All of blood samples and homogenate were centrifuged (3500 rpm/min), and the supernatant was stored at −80°C until analysis. 20 *μ*L of internal standard (Cinobufagin, 200 ng/ml) was added to 100 *μ*L of plasma or supernatant sample. The mixture was vortexed for 0.5 min and 2.5 ml ethylene acetate was added centrifuged at 3500 rpm/min for 10min. Then 2 ml of the supernatant was collected and evaporated to dryness at 45°C and the residue was then redissolved in mobile phase (100 *μ*L). Then, a 10 *μ*L aliquot of supernatant was injected into the HPLC-MS/MS system.

### Antitumor activity *in vitro*

Human hepatoma cell line (SMMC-7721), provided by Shanghai Cellular Institution of Chinese Science Academy, were grown in DMEM containing 10% FBS and kept at 37°C in a humidified incubator with 5% CO_2_ and 97% relative humidity. Cells were plated into a 96-well plate at 1 × 10^4^/well, and cultured routinely for 24 h. 20 *μ*L of different concentrations of Bufalin-BSA-NP and Bufalin solution diluted by DMEM + 10% FBS were added into according ditch in experimental groups. Same volume of tumor cells served as a negative control. There were five pores of each group of each concentration. At 48 h point of culture, 20 *μ*L of MTT was added in and then continued to culture for 4h. The supernatant was discarded and 100 *μ*L of DMSO was added, oscillated until full dissolution, absorption (A) was determined by ELLISA at 570nm. Cell survival was evaluated by the MTT method and is reported as IC_50_, i.e., the concentration (micromoles per liter) of the test compound that inhibits 50% of cell growth. The cell viability was calculated by using the formula: ([ES – BC] / [US – BC]) × 100%. ES, US and BC were defined as the absorbance of experimental samples, untreated samples and blank controls, respectively.

### Antitumor activity *in vivo*

**Figure 7:**
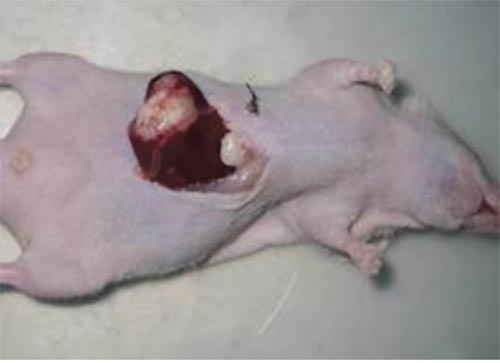
The nude mice orthotopic transplantation tumor model (10 days after transplantation)

The nude mice orthotopic transplantation tumor models were established by using the intrahepatic tunnel implantation [33]. The SMMC-7721 cells (2×10^6^ cells) were injected subcutaneously into nude mice to construct implanted tumors. When the tumor reached diameter of 1 cm it was dissected and cut into pieces of around 2×2×1 mm^3^. These pieces were transplanted into the capsule of the left lateral liver lobe of another nude mouse. The figure of the nude mice orthotopic transplantation tumor model (10 days after transplantation) was showed in Figure 7. After he nude mice orthotopic transplantation tumor models have been made sucessfully, sixty mice were equally randomized into four groups: Bufalin-BSA-NP group, Bufalin group, positive group (ADM group) and negative group (NS group) (n = 15). Bufalin-BSA-NP and Bufalin were injected intravenously at dose of 1 mg/kg for day 14 to day 23 after establishment of the model. NS group were injected equal volume normal saline as above. Adriamycin were injected intravenously into ADM group at dose 8.0 mg/kg at day 14. Ten mice in each group were killed at day24 by cervical dislocation and the tumors tissue were removed and detected the volumes. Five mice in each group were kept alive for observation of tumor-bearing survival. The longest diameter (a) and short diameter (b) of the tumor body were measured by using a slide gaud, and the tumor volume was calculated according the formula V = ab^2^/2; the tumor inhibitory rate = (1 – mean tumor volume of the drug group/mean tumor volume of the control group) × 100%; prolonged survival = (mean days of survival of the drug group/mean days of survival of the control group – 1) × 100%.

### Statistical analysis

Group differences in continuous variables were calculated with Student t-test. One-way ANOVA with the Dunnett's or Newman Keuls post-test was used to compare the means of three or more groups. *P <* 0.05 was considered statistically significant. Data in this study were analyzed by using the software SPSS 21.0 (SPSS Inc., IL, USA).

## Abbreviations

Bufalin-BSA-NP: Bufalin-loaded bovine serum albumin nanoparticle
BSA: Bovine serum albumin
HCC: hepatocellular carcinoma
TCM: traditional Chinese medicine
AUC: area under the curve
MRT: mean residence time
ADM: adriamycin
NS: normal saline
t_1/2_: half-life of plasma
*C*_max_: maximum concentration
Vd: aparent volume of distribution
CL: clearance
RES: reticulate endothelial system
